# Longitudinal Circulating Tumor DNA Profiling in Metastatic Colorectal Cancer During Anti-EGFR Therapy

**DOI:** 10.3389/fonc.2022.830816

**Published:** 2022-02-24

**Authors:** Wentao Yang, Jianling Zou, Ye Li, Rujiao Liu, Zhengqing Yan, Shiqing Chen, Xiaoying Zhao, Weijian Guo, Mingzhu Huang, Wenhua Li, Xiaodong Zhu, Zhiyu Chen

**Affiliations:** ^1^Department of Gastrointestinal Medical Oncology Fudan University Shanghai Cancer Center, Shanghai, China; ^2^Department of Oncology, Shanghai Medical College, Fudan University, Shanghai, China; ^3^Department of Integrative Oncology, Fudan University Shanghai Cancer Center, Shanghai, China; ^4^The Medical Department, 3D Medicines Inc., Shanghai, China

**Keywords:** colorectal cancer, next-generation sequencing, circulating tumor DNA, dynamic monitoring, prognosis

## Abstract

**Background:**

Metastatic colorectal cancer (mCRC) is a heterogenous disease with limited precision medicine and targeted therapy options. Monoclonal antibodies against epidermal growth factor receptor (EGFR) have been a crucial treatment option for mCRC. However, proper biomarkers for predicting therapeutic response remain unknown. As a non-invasive test, circulating tumor DNA (ctDNA) is appropriately positioned to reveal tumor heterogeneity and evolution, as it can be used in real-time genomic profiling. To evaluate the significance of ctDNA in monitoring the dynamic therapeutic response and prognosis of mCRC, we detected the baseline and dynamic changes of ctDNA in mCRC patients receiving anti-EGFR therapies.

**Methods:**

A single-center study was conducted retrospectively. Plasma samples from mCRC patients who received anti-EGFR therapies were collected at baseline and continuous treatment points. The ctDNA was extracted and sequenced with a target panel of tumor-related genes *via* next-generation sequencing (NGS). Clinical information was also collected and analyzed.

**Results:**

We conducted dynamic sampling of 22 mCRC patients, analyzed 130 plasma samples, obtained a baseline genomic mutation profile of the patients. In total, 54 variations were detected in 22 plasma samples, with a positive rate of 77.3% (17/22). TP53 was the most mutated gene (59.1%, 13/22), followed by APC (18.2%, 4/22). There was a high concordance rate of genomic characteristics between the tumor tissue test by polymerase chain reaction and ctDNA test by NGS. The mutation discrepancy increased with an extended course of treatment. During remission TP53 and APC were the most frequently decreased clonal mutations and KRAS, NRAS, ERBB2 and PIK3CA were the most decreased subclonal mutations. Both mutation types were increased during progression. The ctDNA decreased earlier than did the responses of computed tomography and traditional tumor markers (carbohydrate antigen 19-9 and carcinoembryonic antigen [CEA]). Lactate dehydrogenase level *(P* = 0.041), CEA level (*P* = 0.038), and primary lesion site (*P* = 0.038) were independent risk factors that influenced overall survival. Moreover, patients with RAS mutations tended to have a worse prognosis (*P* = 0.072).

**Conclusions:**

This study demonstrates that ctDNA is a promising biomarker for monitoring the dynamic response to treatment and determining the prognosis of mCRC.

## Introduction

Colorectal cancer (CRC) is the third most common cancer and second most frequent cause of cancer-related death worldwide (1). Most CRC patients are diagnosed in an advanced stage; even patients diagnosed in an early stage will develop advanced disease. Palliative chemotherapy has been the mainstay treatment for metastatic CRC (mCRC), and the overall survival (OS) rate of mCRC patients is less than 3 years (2). The emergence and application of targeted therapies have greatly improved OS (3). Cetuximab is a chimeric human/mouse immunoglobulin G1 monoclonal antibody that targets the human epidermal growth factor receptor (EGFR) protein. Several international multicenter clinical studies have shown that cetuximab extends median survival to approximately 30 months in RAS and BRAF wild-type mCRC cases (4–6).

It is important to note that 40–60% of mCRC patients with initial wild-type RAS and BRAF genotypes develop drug resistance after prolonged exposure to cetuximab (7–9). There is evidence that mitogen-activated protein kinase (MAPK) signal transduction pathway-related genes, c-Met gene amplification, and secondary changes of other genes in the human EGF family may be important mechanisms underlying resistance to anti-EGFR monoclonal antibodies (10). Misale et al. (11) showed at both the cellular level and in the clinic that secondary KRAS mutations may be the mechanism responsible for drug resistance following EGFR blockade. Genomic analyses of biopsied tissue after the development of anti-EGFR therapy resistance have shown multiple mutations in KRAS, NRAS, BRAF, and phosphoinositide 3-kinase catalytic subunit alpha (PIK3CA) genes (12, 13). In addition, abnormal changes in genes in the HER family contribute to anti-EGFR resistance. Such changes include mutations in the EGFR extracellular domain and HER-2 amplification, which is common in breast and gastric cancers. Therefore, assessing genomic alterations will help identify potential drug resistance, allowing physicians to adjust treatment decisions in a timely manner.

Circulating tumor DNA (ctDNA) originates from the apoptotic and necrotic turnover of cancer cells. Its genomic profile corresponds with the tumor DNA from which it was derived. Previous studies have shown that ctDNA is enriched in the plasma of cancer patients and its characteristics are representative of the entire tumor genome (14–16). By defining the genomic features in patient plasma with next-generation sequencing (NGS), ctDNA-based liquid biopsy is a convenient, minimally invasive test with reproducible results. It also complements the limitations of tissue evaluation and helps to monitor the molecular changes that occur during cancer evolution (17–19). In addition, the consistency of the RAS (including KRAS and NRAS) gene between tissue samples and liquid biopsy is approximately 93% (20). Therefore, ctDNA detection has been used to make treatment decisions in various types of cancer such as colorectal, lung, and gastroesophageal cancers (21–23).

Previous studies have revealed the importance of ctDNA in CRC. For patients who undergo surgery, ctDNA levels have been used to detect minimal residual disease and predict prognosis (24, 25). Moreover, the use of ctDNA in dynamic monitoring of the treatment response has been actively explored. There is growing evidence supporting the significance of ctDNA in the therapeutic response and drug resistance. Detectable ctDNA levels at baseline and new emerging ctDNA at follow-up treatment are associated with a poor prognosis (26). Moreover, decreased ctDNA levels reflect sensitivity to anti-EGFR therapies (27, 28). However, more studies are needed to identify the ctDNA features and dynamic changes in mCRC patients.

In this study, we identified ctDNA profiling at baseline and dynamic changes during anti-EGFR treatments. We also explored the relationships between ctDNA abundance and clinical characteristics, prognosis, and therapeutic evaluation.

## Materials and Methods

### Patients

Patients pathologically diagnosed with mCRC at Fudan University Shanghai Cancer Center (Shanghai, China) from October 12, 2016 to March 20, 2020 were included in this study retrospectively. The inclusion criteria were as follows: diagnosis of mCRC; presence of at least one measurable or unmeasurable but evaluable lesion (described according to Response Evaluation Criteria in Solid Tumors [RECIST] 1.1); presence of polymerase chain reaction (PCR)-confirmed wild-type KRAS (exon 2/3/4), NRAS (exon 2/3/4), and BRAF (exon 15) genotypes in tumor tissue before the receipt of anti-EGFR therapy; no history of severe heart or liver disease, psychiatric disorders, hemorrhage, or perforation of the digestive tract; and an Eastern Cooperative Oncology Group performance status of 0/1 at 3 days before treatment. Exclusion criteria were as follows: presence of mCRC combined with other types of cancer. The study was conducted in accordance with the Declaration of Helsinki (as revised in 2013). The study protocol was approved by the Ethics Committee of Fudan University Shanghai Cancer Center (Shanghai, China). All patients provided written informed consent to participate. Tumor burden was measured to evaluate the clinical response by computed tomography (CT) or magnetic resonance imaging (MRI) according to RECIST 1.1. Each patient’s response to anti-EGFR therapy was recorded as partial response, stable disease, or progressive disease according to RECIST 1.1 criteria. No complete responses were observed in this cohort. Progression-free survival (PFS) was defined from the date informed consent was provided until the evaluation of progressive disease. OS was defined from the date informed consent was provided until the day of death or the last day of follow-up.

### Blood Sample Processing and ctDNA Isolation

Peripheral blood (5–10 mL) was collected at baseline and at 2-month intervals during treatment. The blood samples were centrifuged in Streck tubes at 1,600 x g at 4°C for 10 min. The supernatants were transferred to new tubes and stored at -80°C before use. ctDNA was extracted using the QiAmp Circulating Nucleic Acid Kit (Qiagen, Germantown, MD, USA) in accordance with the manufacturer’s instructions. DNA concentrations were quantified with the Qubit dsDNA HS Assay Kit (Thermo Fisher Scientific, Waltham, MA, USA). ctDNA was extracted and sequenced with a target panel of 61 genes (**Supplementary Table 1**) in a laboratory that was certified by both the College of American Pathologists and Clinical Laboratory Improvement Amendments.

### Targeted Capture Sequencing

Cell-free DNA libraries were prepared using the KAPA Hyper Prep Kit (KAPA Biosystems Inc., Wilmington, MA, USA) in accordance with the manufacturer’s protocol. They were individually barcoded with unique molecular identifiers. In brief, 30–60 ng ctDNA were subjected to end-repairing, A-tailing, and ligation with indexed adapters. Then, the libraries were PCR-amplified and purified for target enrichment. The concentration and size distribution of each library were determined using a Qubit 3.0 fluorometer (Thermo Fisher Scientific) and a LabChip GX Touch HT Analyzer (PerkinElmer, Waltham, MA, USA), respectively.

For targeted capture, indexed libraries were subjected to probe-based hybridization with a customized NGS panel that included 61 cancer-related genes. The probe baits were used to individually synthesize 5′ biotinylated 120 base pair (bp) DNA oligonucleotides (IDT, Coralville, IA, USA). Repetitive elements were filtered out from intronic baits according to annotations by UCSC Genome RepeatMasker (29). The xGen^®^ Hybridization and Wash Kit (IDT) was employed for hybridization enrichment. Briefly, 500 ng indexed DNA libraries were pooled to obtain 2 μg DNA. Pooled DNA samples were mixed with Human Cot-1 DNA and xGen Universal Blockers-TS Mix and dried in a SpeedVac system. Hybridization Master Mix was added to each sample. The mixtures were incubated in a thermal cycler at 95°C for 10 min, then combined with 4 μL probes and incubated at 65°C overnight. Target regions were captured in accordance with the manufacturer’s instructions. The concentration and fragment size distribution of the final library were determined using a Qubit 3.0 fluorometer (Thermo Fisher Scientific) and LabChip GX Touch HT Analyzer (PerkinElmer), respectively. The captured libraries were loaded onto a NovaSeq 6000 platform (Illumina, San Diego, CA, USA) for 100 bp paired-end sequencing with a mean sequencing depth of 36000.

Raw data were mapped to the reference human genome hg19 using the Burrows-Wheeler Aligner. In-house developed software was used to generate duplex consensus sequences based on dual unique molecular identifiers integrated at the ends of the DNA fragments. To improve specificity, particularly for variants with low allele frequency in the ctDNA, an in-house loci-specific variant detection model based on a binomial test was applied. The variants were subsequently filtered according to their supporting count, strand bias status, base quality, and mapping quality. In addition, variant calling was optimized to detect variants in short tandem repeat regions. Single-nucleotide polymorphisms (SNPs) and indels were annotated by ANNOVAR against the following databases: dbSNP (v138), 1000Genome, and ESP6500 (population frequency > 0.015). Only missense, stop-gain, frameshift, and non-frameshift indel mutations were kept. Copy number variations and gene rearrangements were detected as described previously (30). We calculated the sum of the variant allele frequency (VAF) for each sample. In this manner, the sum of VAF in percentages represented most of the ctDNA detected at each time point. Because there are no established cutoffs for clinically significant treatment-induced changes in ctDNA, we predefined molecular progression as an increase in the mean VAF by at least 25% or new emerging variant allele if the VAF was negative at baseline. We predefined molecular remission as a decrease in the mean VAF by at least 50% for patients in whom the VAF was positive at baseline. A mutation was defined as “subclone” if the VAF was less than 25% of the highest in the sample or as “clone” if the VAF was above this threshold, according to the method used in a previous study (15).

### Statistical Analyses

Numerical diversity between subgroups was assessed using the Wilcoxon–Mann–Whitney test. Survival results were assessed by Kaplan–Meier survival analysis paired with the log-rank test and Cox proportional hazards modeling. For all tests, *P* < 0.05 was considered statistically significant. All statistical analyses were performed using SPSS software (v. 21.0; SPSS Inc., Chicago, IL, USA) or GraphPad Prism (v. 8.0; La Jolla, CA, USA).

## Results

### Characteristics of Patients

From October 12, 2016 to March 20, 2020, 22 mCRC patients were enrolled in this trial (**Supplementary Figure 1**). Patient characteristics are shown in **Table 1**. There were 18 men and 4 women, all of whom were treated with cetuximab and standard chemotherapy. The median age was 61 years (31–73 years); 81.8% of patients (18/22) had left-sided CRC and 18.2% (4/22) had right-sided CRC. Half of the patients had undergone primary tumor resection at baseline. Twenty-one (21/22, 95.5%) patients had synchronous metastasis and one (1/22, 4.5%) patient had metachronous metastasis. Furthermore, 68.2% (15/22) of the patients had liver metastasis at baseline, 13.6% (3/22) had lung metastasis, 9.1% (2/22) had peritoneum metastasis, and 22.7% (5/22) had distant lymph node metastasis. In total, 68.2% (15/22) of the patients received cetuximab as first-line therapy, 27.3% (6/22) received cetuximab as second-line therapy, and 4.5% (1/22) received cetuximab as third-line therapy. The median serum lactate dehydrogenase (LDH) level was 204.5 U/L (range: 135.0–3000.0 U/L), and the median serum carcinoembryonic antigen (CEA) level was 35.2 ng/mL (range: 2.0–1000.0 ng/mL).

**Table 1:** Baseline patients’ characteristics (n=22^a^).

Characteristics	N (%)
Age (mean and range)	61 (31-73)
≤60	10 (45.5)
>60	12 (54.5)
Gerder	
Male	18 (81.8)
Female	4 (18.2)
Primary tumor resection	
Yes	11 (50.0)
No	11 (50.0)
Anatomical position of primary lesion	
Left	18 (81.8)
Right	4 (18.2)
Differentiation	
Well	0 (0)
Moderate	14 (63.6)
Poor	6 (27.3)
Unknown	2 (9.1)
Onset of metastasis	
Synchronous metastasis	21 (95.5)
Metachronous metastasis	1 (4.5)
Histological type	
Adenocarcinoma	19 (86.4)
Mucinous carcinoma	1 (4.5)
Adenocarcinoma with mucinous component	2 (9.1)
Serum LDH level (U/L, median and range)	204.5 (135.0-3000.0)
<240	11 (50.0)
≥240	11 (50.0)
Serum CEA level (ng/ml, median and range)	35.2 (2.0-1000.0)
<5.4	5 (22.7)
≥5.4	17 (77.3)
Metastatic sites involved	
≤1	13 (59.1)
>1	9 (40.9)
Metastatic site	
Liver	15 (68.2)
Distant lymph nodes	5 (22.7)
Lung	3 (13.6)
Peritoneum	2 (9.1)
Others	5 (22.7)
Cetuximab use as	
1st line	15 (68.2)
2nd line	6 (27.3)
3rd line	1 (4.5)
Combined chemotherapy	
FOLFOX	14 (63.6)
FOLFIRI	6 (27.3)
XELOX	1 (4.5)
Irinotecan	1 (4.5)

a Overall 22 patients were enrolled for the study.

### Mutation Profiles at Baseline

**Figure 1:**
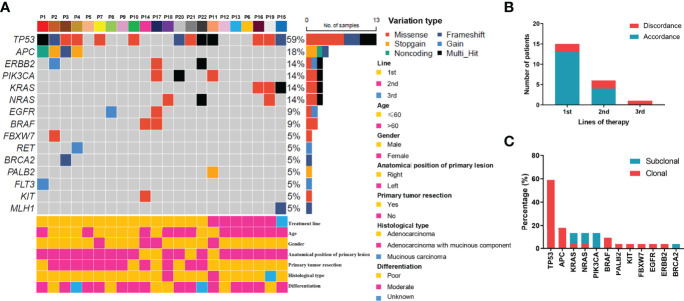
Mutation profiling of pre-treatment ctDNA. **(A)** Genomic profiles of 22 advanced colorectal cancer patients from pre-treatment ctDNA. **(B)** The consistency of the RAS mutations detected in paired tissues and plasma. **(C)** The clonal and subclonal landscapes in 22 mCRC patient at baseline. Gain: segments with log ratio more than 3 times of standard deviation of all segment level were considered as “gain”.

For the 22 enrolled patients, ctDNA was extracted from 130 plasma samples and sequenced by NGS. The genomic features of 61 genes (**Tables S1**), including copy number variations and mutations, were evaluated as treatment proceeded. The mutation profiles at baseline are shown in **Figure 1**. Using targeted capture sequencing, 54 variations were detected in 22 plasma samples, with a positive rate of 77.3% (17/22). Fifteen genes with different types of variation were identified, including missense, frameshift, stop-gain, gain, noncoding, and multiple variations (**Figure 1A**). Tumor protein 53 (TP53) was the most mutated gene (59.1%, 13/22), involving seven missense mutations, three frameshift mutations, and three multiple variations. Other mutated genes were APC (18.2%, 4/22), NRAS (13.6%, 3/22), KRAS (13.6%, 3/22), Erb-B2 receptor tyrosine kinase 2 (ERBB2) (13.6%, 3/22), and PIK3CA (13.6%, 3/22).

For the KRAS, NRAS, and BRAF V600E genes, the concordance rates between the tumor tissue test by PCR and ctDNA test by NGS were 86.4%, 86.4%, and 100%, respectively. The RAS mutation discrepancy was also compared among treatments (**Figure 1B**). For patients who received cetuximab as first-line treatment, the RAS mutation discrepancy was 13.3% (2/15). Both of these patients also had NRAS mutations. The mutation sites were NRAS p.Q61K (0.31%), NRAS p.G13R (0.07%), and NRAS p.G12R (0.37%). For patients who received cetuximab as second-line treatment, the RAS mutation discrepancy was 33.3% (2/6). One patient had a KRAS p.G12V mutation (2.17%) and the other patient had both KRAS p.Q61H (0.02%) and NRAS p.G13C (0.03%) mutations. The only patient who received cetuximab as third-line treatment had a KRAS mutation. The mutation sites included KRAS p.Q61Hc.183A>T (0.05%), KRAS p.Q61Hc.183A>C (0.91%), and KRAS p.G12A (0.58%). The clonal and subclonal landscapes were detected at baseline (**Figure 1C**). Subclonal mutations were found in 31.8% (7/22) of the patients. The three most common clonal mutation genes were TP53, APC, and BRAF, while the three most common subclonal mutation genes were PIK3CA, KRAS, and NRAS.

### Dynamic Changes in Mutations During Treatment

**Figure 2:**
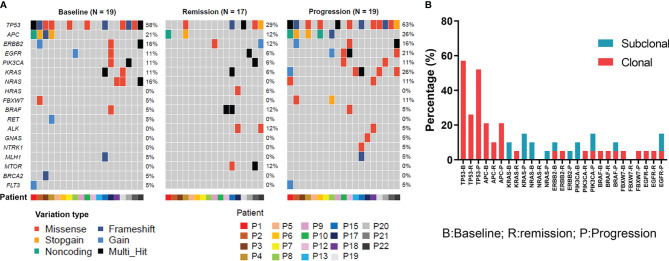
Genomic features of ctDNA dynamically changed in colorectal cancer patients received anti-EGFR therapies. **(A)** The ctDNA genomic features for all specimens at baseline, remission, and progression in colorectal cancer patients who received cetuximab-targeted therapy. **(B)** The clonal and subclonal landscapes in mCRC patient at baseline, remission, and progression. Gain: segments with log ratio more than 3 times of standard deviation of all segment level were considered as “gain”.

To evaluate the dynamic changes in mutations during treatment, the patients’ genomic landscapes of baseline, optimal remission, and progression are shown in **Figure 2A**. Three patients had unavailable plasma during disease progression and an additional two patients had unavailable plasma during remission. Thus, the dynamic changes were analyzed in 19 patients at baseline and progression, while they were analyzed in 17 patients at remission. Overall, compared with baseline, the gene alterations in ctDNA were decreased during remission. These reductions included TP53 (29% vs. 58%), APC (12% vs. 21%), ERBB2 (12% vs. 16%), PIK3CA (6% vs. 11%), KRAS (6% vs. 11%), and NRAS (0% vs. 16%). In contrast, ctDNA appeared again or the corresponding number of gene alterations was increased during progression. These changes involved TP53 (29% vs. 63%), APC (12% vs. 26%), ERBB2 (12% vs. 16%), PIK3CA (6% vs. 11%), KRAS (6% vs. 26%), and NRAS (0% vs. 11%). These results indicate that ctDNA could possibly be used as an alternative tool for evaluating treatment efficacy. Moreover, dynamic detection revealed that TP53 and APC were the most frequently decreased clonal mutations during remission, while they were increased during progression (**Figure 2B**). KRAS, NRAS, ERBB2, and PIK3CA were the most decreased subclonal mutations during remission. Similarly, they were increased during progression.

**Figure 3:**
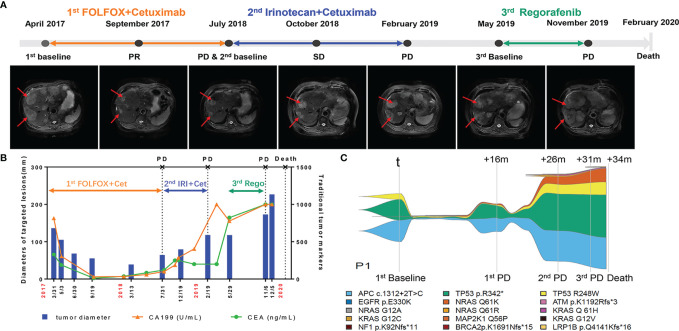
Longitudinal radiograph and ctDNA detection of one representative CRC patient. **(A)** Radiographic features of tumor lesions in a 73-year-old male with colorectal cancer liver metastasis. PR, partial response; SD, stable disease; PD, progressive disease. **(B)** Comparison of the changes of tumor diameter, CEA and CA199 in patient 1(P1). Cet: Cetuximab; IRI, Irinotecan; Rego, Regorafenib. **(C)** Example of a serial ctDNA testing in P1 showing emergence of clonal alterations with treatment process. The changes of line thickness indicate the changes of variant allele frequency (VAF) of genomic alterations. The thicker line corresponds to larger VAF. Colors of specific genomic alterations shown on the bottom.

To further evaluate the consistency of the gene mutation profile in ctDNA and clinical parameters, the following is a description of a typical case. This patient (P1) had a primary tumor on the left side with synchronous liver metastasis and tumor resection before receiving cetuximab. Throughout the course of treatment, this patient received three different lines of treatment. For the first line, he received cetuximab in combination with FOLFOX for 6 months and cetuximab in combination with leucovorin and 5-fluorouracil for another 10 months, and then got progressive disease and changed to second line therapy. This patient declined intensive chemotherapy including venous 5-fluorouracil at that time, so he received cetuximab in combination with irinotecan as second-line treatment. After progression at February 2019, he refused any treatment. And then he began to receive oral regorafenib as third line treatment at May 2019. He did not received bevacizumab in his therapeutic process for the financial reason. **Figure 3A** illustrates the overall treatment procedure in P1 and the corresponding lesions on CT. The changes in tumor diameter and tumor antigen biomarkers (carbohydrate antigen 19-9 [CA19-9] and CEA) are shown in **Figure 3B**. **Figure 3C** illustrates the serial ctDNA testing in P1, showing the emergence of clonal alterations through the treatment process. The patient had APC c.1312+2T>C, TP53 p.R342, and EGFR p.E330K, which were considered clonal alterations at baseline. He developed KRAS and NRAS mutations when progressing after 8 months of treatment with cetuximab. However, APC c.1312+2T>C and EGFR p.E330K remained the most frequently altered genes. After the application of second-line therapy, stable disease was achieved. The decrease in mutation frequency was consistent with clinical efficacy. The mutation frequency then increased, followed by progressive disease assessed by CT scan. We observed an increase in existing mutated genes, as well as the emergence of new gene alterations in the EGFR pathway (e.g., MAP2K [Dual Specificity Mitogen-Activated Protein Kinase], EGFR [Epidermal Growth Factor Receptor], ATM [ATM Serine/Threonine Kinase], BRCA2 [Breast Cancer Type 2 Susceptibility Protein], NF1 [Neurofibromin 1], and LRP1B [LDL Receptor Related Protein 1B] mutations) until the patient’s death.

As shown in **Figure S2**, the ctDNA decreased to a very low level in June 2017, which was 3 months earlier than the responses of CT and traditional tumor markers (CA199 and CEA). The first progressive disease was observed in July 2018. In contrast, increased ctDNA was detected in May 2018, which was 2 months earlier than the CT results. These results support the use of ctDNA for evaluating treatment efficacy in advanced CRC cases.

### Association Between Genomic Features of ctDNA and Prognosis

To investigate the significance of ctDNA in prognosis, the associations of genomic features of ctDNA with PFS and OS were analyzed. For PFS, univariate survival analysis revealed that the histological type *(P* = 0.045) and RAS status at baseline (*P* = 0.002) were poor prognostic indicators (**Table 2**). For OS, univariate survival analysis revealed that LDH level (*P* = 0.047), CEA level (*P* = 0.008), metastasis sites involved (*P* = 0.012), histological type (*P* = 0.003), and RAS status at baseline (*P* = 0.002) were poor prognostic indicators (**Table 3**). The variable factors, including LDH level, CEA level, metastatic sites involved, histological type, differentiation, mean VAF, primary lesion site, absence or presence of primary tumor resection, and RAS status at baseline, were included in the multivariate analyses. Although no factor was associated with PFS, LDH level (*P* = 0.041), CEA level (*P* = 0.038), and primary lesion site (*P* = 0.038) were independent risk factors. In addition, patients with a RAS mutation tended to have a worse prognosis (*P* = 0.072).

**Table 2:** Univariate and multivariate analysis of progression free survival (n=22).

Variable	Univariate analysis				Multivariate analysis			
	B		Exp (B) (95% CI)	P	B		Exp (B) (95% CI)	P
LDH, U/L	-0666		0.514 (0.204-1.295)	0.158	-0.312		0.732 (0.075-7.169)	0.789
CEA, ng/ml	-1.203		0.300 (0.082-1.096)	0.085	-1.064		0.345 (0.024-4.948)	0.434
Metastatic sites involved	0.931		2.538 (0.936-6.884)	0.067	0.707		2.027 (0.331-12.420)	0.445
Histological type	1.874		6.512 (1.047-40.524)	0.045	1.168		3.216 (0.084-122.749)	0.530
Differentiation	-1.008		0.365 (0.128-1.039)	0.059	-0.615		0.541 (0.095-3.069)	0.488
Mean VAF	-0.120		0.887 (0.321-2.452)	0.887	-0.801		0.449 (0.056-3.574)	0.449
Site of primary lesion	0.382		1.466 (0.418-5.38)	0.550	-0.173		0.841 (0.087-8.124)	0.881
Primary tumor resection	-0.119		0.888 (0.366-2.152)	0.793	-0.013		0.987 (0.153-6.362)	0.989
RAS status at baseline	2.029		7.610 (2.093-27.669)	0.002	0.921		0.987 (0.153-8.362)	0.443

LDH, lactate dehydrogenase; CEA, carcinoembryonic antigen.

**Table 3:** Univariate and multivariate analysis of overall survival (n=22).

Variable	Univariate analysis				Multivariate analysis			
	B		Exp (B) (95% CI)	P	B		Exp (B) (95% CI)	P
LDH, U/L	-1.196		0.302 (0.093-0.987)	0.047	-5.740		0.003 (0-0.788)	0.041
CEA, ng/ml	-1.743		0.175 (0.049-0.629)	0.008	-9.153		0 (0-0.599)	0.038
Metastatic sites involved	1.403		4.069 (1.364-12.138)	0.012	3.058		21.277 (0.555-815.122)	0.100
Histological type	3.429		30.854 (3.106-306.456)	0.003	28.994		3.907E+12 (0-9.18E+48)	0.856
Differentiation	-0.497		0.608 (0.182-2.036)	0.420	5.141		170.811 (0.072-403150.683)	0.195
Mean VAF	1.269		3.559 (0.790-16.023)	0.098	1.791		5.994 (0.174-206.541)	0.321
Site of primary lesion	-0.552		0.576 (0.157-2.118)	0.406	-5.162		0.006 (0-0.933)	0.047
Primary tumor resection	-0.091		0.913 (0.39-2.618)	0.886	2.795		16.365 (0.569-470.439)	0.103
RAS status at baseline	2.044		7.721 (2.120-28.124)	0.002	-11.995		0 (0-2.945)	0.072

LDH, lactate dehydrogenase; CEA, carcinoembryonic antigen.

## Discussion

Considering the growing evidence that ctDNA sequencing could represent a valuable resource for genomic discovery, we analyzed the ctDNA profiles of 22 CRC patients from a single-center retrospectively. We found a high similarity of genomic alterations in ctDNA and tumor tissue, similar to that described in a previous report (31). We also identified the baseline characteristics and dynamic changes in ctDNA mutations during anti-EGFR treatment. These results suggest that ctDNA is a stable biomarker available for auxiliary clinical diagnosis, as well as for evaluating CRC tumor progression.

Currently, CT and MRI scans are recommended as the main diagnostic and surveillance methods for mCRC patients (32). However, only enlarged tumors can be identified in this manner. Such tumors always exhibit drug resistance, which limits treatment effectiveness (33). Thus, predictive and prognostic markers that represent therapeutic resistance at an early stage are urgently needed. CEA and LDH are often used for auxiliary diagnosis and therapeutic evaluation. Yet, they are insufficient for reflecting genomic alterations. Thus, there is an unmet clinical need for a biomarker that more accurately reflects therapeutic efficacy and dynamic changes during therapy. Liquid biopsy, particularly ctDNA from plasma, has high sensitivity and specificity in early cancer detection (34), so it may be useful for assessing dynamic changes in genes. Furthermore, because tumor tissue sequencing often relies on archival tissue obtained prior to the development of metastatic disease, ctDNA profiling may more readily facilitate the analysis of patients with metastatic disease by better capturing the presence of tumor heterogeneity.

Our study focused mainly on therapeutic resistance. Because tissue-based sequencing compendia depend mainly on treatment-naïve tumors in the early stages of development, the results generally cannot focus on acquired resistance. Conversely, ctDNA sequencing can more easily provide non-invasive access to patients with advanced tumors and offer unique insights into resistance mechanisms that emerge under the selective pressures of different therapies. Our analysis identified clonal and subclonal gene mutations, the frequencies of which were decreased during remission and increased during progression. These results confirmed that the recurrent alterations of these previously identified gene alterations in ctDNA were decreased during remission. In contrast, the ctDNA appeared again or the corresponding number of gene alterations was increased during progression, which is possibly associated with tumor progression.

Notably, the acquired mutations of KRAS or NRAS can be detected in the ctDNA of mCRC patients who initially exhibited wild-type genotypes and thus received anti-EGFR therapies. The mutation frequency fluctuated in a dynamic manner according to treatment efficacy, consistent with our dynamic monitoring results. After changes to the secondary therapeutic approach without anti-EGFR antibodies, the mutation frequency can greatly decrease and may disappear. This provides an opportunity to re-challenge the tumor with anti-EGFR antibodies. Some retrospective studies have shown that patients with wild-type RAS and BRAF genotypes can benefit such re-challenging (35). Furthermore, the CAVE study demonstrated that cetuximab-based re-challenge therapy in RAS wild-type mCRC, according to the results of ctDNA analysis, could be used for patient selection and may improve OS (36). We are also performing a prospective phase II study to evaluate the significance of ctDNA for treatment decision-making in patients with mCRC after failed first-line cetuximab treatment (NCT04831528).

This study has some limitations. First, the small number of patients included may have diluted the importance of ctDNA as a predictive and prognosis marker in mCRC. According to a previous study, ctDNA has an important role in advanced solid tumors and can be used to predict treatment efficacy in perioperative CRC (15, 22). It could also be a significant marker in mCRC. This limitation can be overcome by validation in a larger study. Second, genomic alterations in ctDNA were not detected in 15% of cases, which was similar to the rates of ctDNA detection in other CRC series (37). Some patients may not have had alterations in genes covered by the NGS assay. However, in most cases, the lack of detected genomic alterations in ctDNA was generally caused by other factors, including low tumor burden, absence of ctDNA shedding by some tumors, and timing of blood collection. Some other approaches, such as multiomics-like methylation, exosomes, circulating microRNA, metabonomics, and/or molecular imaging methods (38, 38), could possibly be used to detect ctDNA at lower thresholds with greater accuracy and provide more practical value for ctDNA detection.

## Conclusion

This study demonstrates that ctDNA may be a reliable biomarker to assist in the prognostic evaluation and assessment of treatment efficacy in advanced CRC patients. Assessments of dynamic changes in ctDNA in mCRC patients can identify baseline values for prognostic evaluation and help with clinical decision-making.

## Data Availability Statement

The data presented in the study are deposited in the Biological ProjectLibrary repository, accession number PRJCA008093, https://ngdc.cncb.ac.cn/bioproject/browse/PRJCA008093

## Ethics Statement

The studies involving human participants were reviewed and approved by the Ethics Committee of Fudan University Shanghai Cancer Center. The patients/participants provided their written informed consent to participate in this study.

## Author Contributions

WY contributed to the data collection, data analysis and writing the manuscript. JZ assisted in data analysis and editing this manuscript. YL assisted in data analysis and data collection. RL and XYZ assisted in data collection. ZY and SC were mainly responsible for genetic testing of samples. WG, WL, MH and XDZ offered part of cases. ZC contributed to the research design, sample collection and manuscript revisions. All authors contributed to the article and approved the submitted version.

## Funding

This project was sponsored by Shanghai Municipal Science and Technology Major Project (19411970800) and Shanghai Sailing Program (20YF1408900).

## Conflict of Interest

Authors ZY and SC were employed by the company 3D Medicines Inc., Shanghai, China.

The remaining authors declare that the research was conducted in the absence of any commercial or financial relationships that could be construed as a potential conflict of interest.

## Publisher’s Note

All claims expressed in this article are solely those of the authors and do not necessarily represent those of their affiliated organizations, or those of the publisher, the editors and the reviewers. Any product that may be evaluated in this article, or claim that may be made by its manufacturer, is not guaranteed or endorsed by the publisher.
