# Corrigendum: Longitudinal Circulating Tumor DNA Profiling in Metastatic Colorectal Cancer During Anti-EGFR Therapy

**DOI:** 10.3389/fonc.2022.903586

**Published:** 2022-05-11

**Authors:** Wentao Yang, Jianling Zou, Ye Li, Rujiao Liu, Zhengqing Yan, Shiqing Chen, Xiaoying Zhao, Weijian Guo, Mingzhu Huang, Wenhua Li, Xiaodong Zhu, Zhiyu Chen

**Affiliations:** ^1^Department of Gastrointestinal Medical Oncology, Fudan University Shanghai Cancer Center, Shanghai, China; ^2^Department of Oncology, Shanghai Medical College, Fudan University, Shanghai, China; ^3^Department of Integrative Oncology, Fudan University Shanghai Cancer Center, Shanghai, China; ^4^The Medical Department, 3D Medicines Inc., Shanghai, China

**Keywords:** colorectal cancer, next-generation sequencing, circulating tumor DNA, dynamic monitoring, prognosis

In the original article, there was a mistake in **Figure 1** as published. The figure illustration is wrong due to a software bug during picture output. The corrected **Figure 1** appears below.

**Figure 1:**
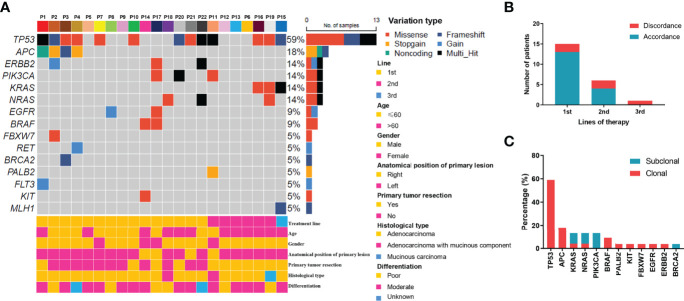
Mutation profiling of pre-treatment ctDNA. **(A)** Genomic profiles of 22 advanced colorectal cancer patients from pre-treatment ctDNA. **(B)** The consistency of the RAS mutations detected in paired tissues and plasma. **(C)** The clonal and subclonal landscapes in 22 mCRC patient at baseline. Gain: segments with log ratio more than 3 times of standard deviation of all segment level were considered as “gain”.

In the original article, references **14** and reference **37** are the same. We have renumbered the references.

In the original article, there was an additional error in **Results, Mutation Profiles at Baseline**, paragraph two. In the sentence “For patients who received cetuximab as second-line treatment, the RAS mutation discrepancy was 13.3% (2/6)”, the percentage is wrong and needs to be changed from 13.3% to 33.3%. The corrected paragraph appears below.

“For the KRAS, NRAS, and BRAF V600E genes, the concordance rates between the tumor tissue test by PCR and ctDNA test by NGS were 86.4%, 86.4%, and 100%, respectively. The RAS mutation discrepancy was also compared among treatments (**Figure 1B**). For patients who received cetuximab as first-line treatment, the RAS mutation discrepancy was 13.3% (2/15). Both of these patients also had NRAS mutations. The mutation sites were NRAS p.Q61K (0.31%), NRAS p.G13R (0.07%), and NRAS p.G12R (0.37%). For patients who received cetuximab as second-line treatment, the RAS mutation discrepancy was 33.3% (2/6). One patient had a KRAS p.G12V mutation (2.17%) and the other patient had both KRAS p.Q61H (0.02%) and NRAS p.G13C (0.03%) mutations. The only patient who received cetuximab as third-line treatment had a KRAS mutation. The mutation sites included KRAS p.Q61Hc.183A>T (0.05%), KRAS p.Q61Hc.183A>C (0.91%), and KRAS p.G12A (0.58%). The clonal and subclonal landscapes were detected at baseline (**Figure 1C**). Subclonal mutations were found in 31.8% (7/22) of the patients. The three most common clonal mutation genes were TP53, APC, and BRAF, while the three most common subclonal mutation genes were PIK3CA, KRAS, and NRAS.”

The authors apologize for these errors and state that this does not change the scientific conclusions of the article in any way. The original article has been updated.

## Publisher’s Note

All claims expressed in this article are solely those of the authors and do not necessarily represent those of their affiliated organizations, or those of the publisher, the editors and the reviewers. Any product that may be evaluated in this article, or claim that may be made by its manufacturer, is not guaranteed or endorsed by the publisher.

## Supplementary Material

The Supplementary Material for this article can be found online at: https://www.frontiersin.org/articles/10.3389/fonc.2022.903586/full#supplementary-material
