# Supplementary material for: Longitudinal Circulating Tumor DNA Profiling in Metastatic Colorectal Cancer During Anti-EGFR Therapy
Source: Front Oncol. 2022 Feb 24;12:830816. doi: 10.3389/fonc.2022.830816 (PMC8908369; doi:10.3389/fonc.2022.830816)
Supplement: Supplementary file 1 [file DataSheet_1.pdf]

Supplementary Material

Supplementary Figures and Tables

1.1 Supplementary Figures

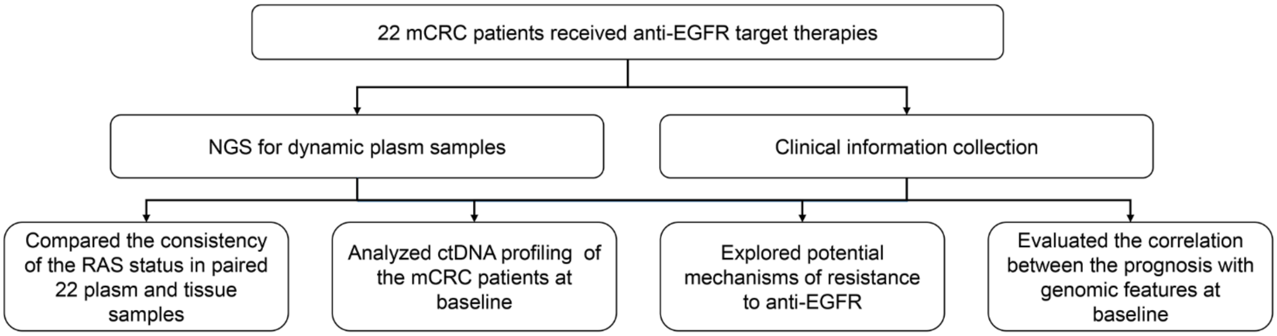

Supplementary Figure 1. An overview of the workflow in this study.

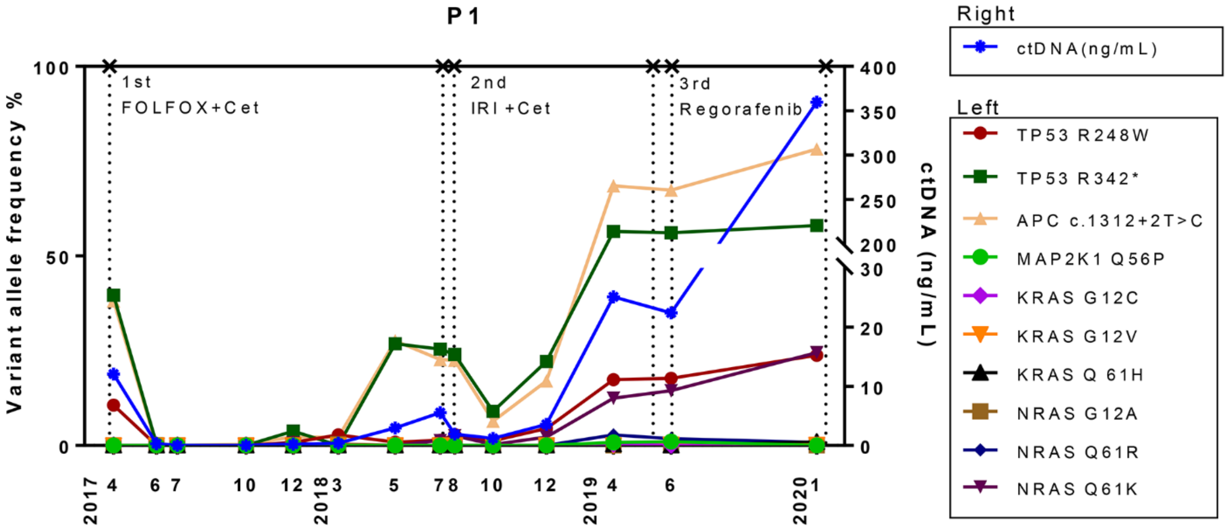

Supplementary Figure 2. Clinical utility of longitudinal ctDNA testing in patient 1 (P1). ctDNA (ng/mL)=VAFmax*m(cfDNA)/Volume

1.2 Supplementary Tables

Table S1. The list of 61 cancer-related genes.

AKT1	ALK	AR	ARAF	BCL2L11	BRAF	BRCA1	BRCA2	CDK4	CDKN2A
CYP2C19	CYP2D6	DPYD	EGFR	ERBB2	ERBB3	ESR1	FGFR1	FGFR2	FGFR3
GNA11	GNAQ	GNAS	HRAS	IDH1	IDH2	JAK2	JAK3	KIT	KRAS
MAP2K1	MAP2K2	MDM2	MET	MLH1	MSH2	MSH6	MTOR	NRAS	NRG1
NTRK1	NTRK2	NTRK3	PALB2	PDGFRA	PDGFRB	PIK3CA	PMS2	POLD1	POLE
PTEN	RAF1	RB1	RET	ROS1	SMO	STK11	TERT	TP53	TPMT
UGT1A1									

Table S2. The list of 65 cancer-related genes.

AKT1	ALK	APC	ARAF	ARID1A	ATM	BCL2L11	BRAF	BRCA1	BRCA2
CCND1	CD274	CDK4	CDK6	CDKN2A	CTNNB1	CYP2C19	DDR2	DPYD	EGFR

ERBB2	ERBB3	FBXW7	FGF19	FGFR1	FGFR2	FGFR3	FLT3	HRAS	JAK2
KDR	KIT	KRAS	LRP1B	MAP2K1	MAP2K2	MET	MLH1	MSH2	MSH6
MTOR	NF1	NRAS	NTRK1	NTRK2	PDCD1LG2	PDGFRA	PIK3CA	PMS2	POLD1
POLE	PTEN	RAF1	RET	ROS1	SMAD4	SMO	SPTA1	STK11	TP53
TPMT	TSC1	TSC2	UGT1A1	VEGFA					
