# Supplementary material for: Corrigendum: Longitudinal Circulating Tumor DNA Profiling in Metastatic Colorectal Cancer During Anti-EGFR Therapy
Source: Front Oncol. 2022 May 11;12:903586. doi: 10.3389/fonc.2022.903586 (PMC9132584; doi:10.3389/fonc.2022.903586)
Supplement: Supplementary file 1 [file DataSheet_1.docx]

Supplementary Material

# Supplementary Figures and Tables

## Supplementary Figures


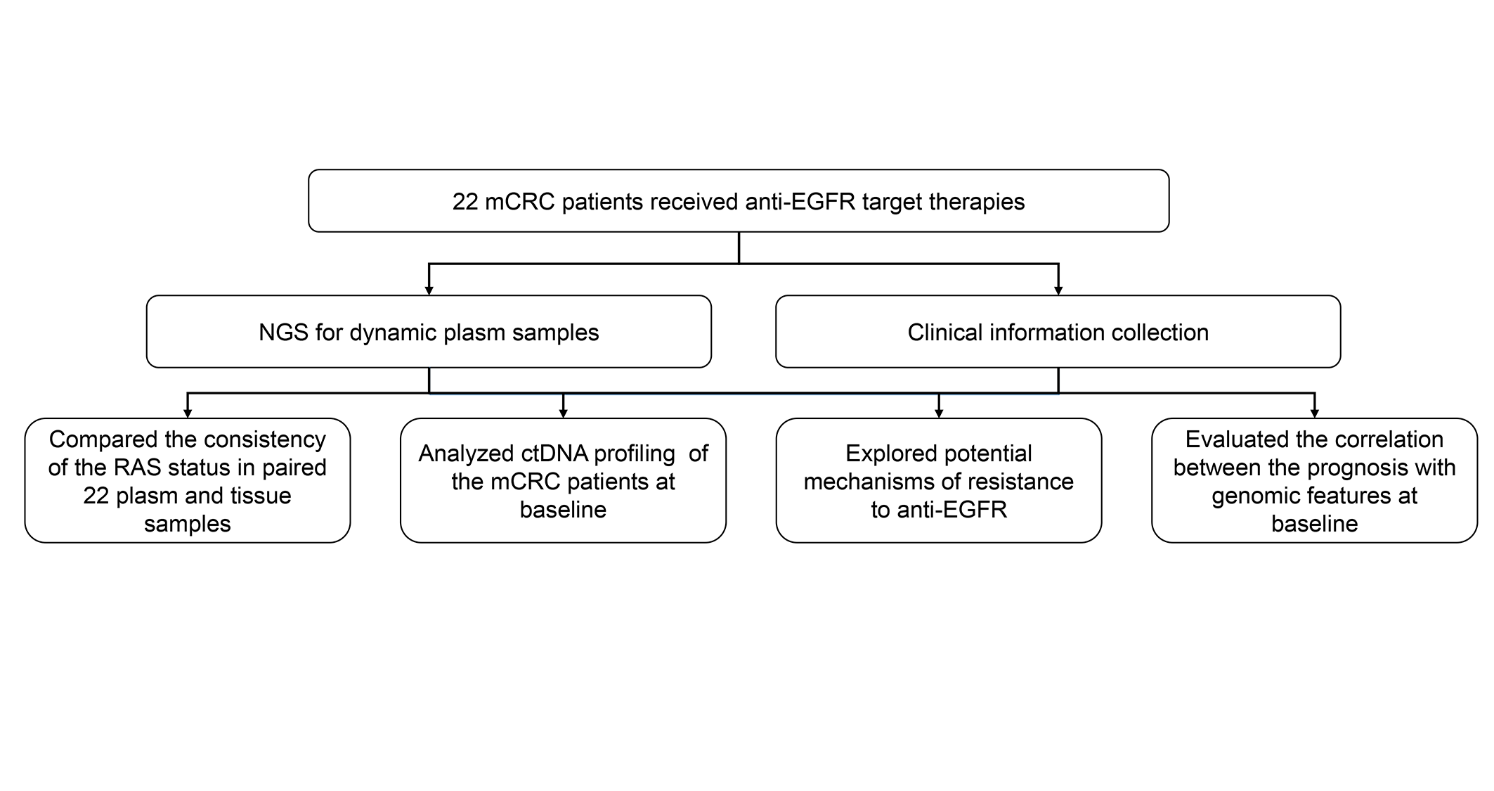
**Supplementary Figure 1.** An overview of the workflow in this study.


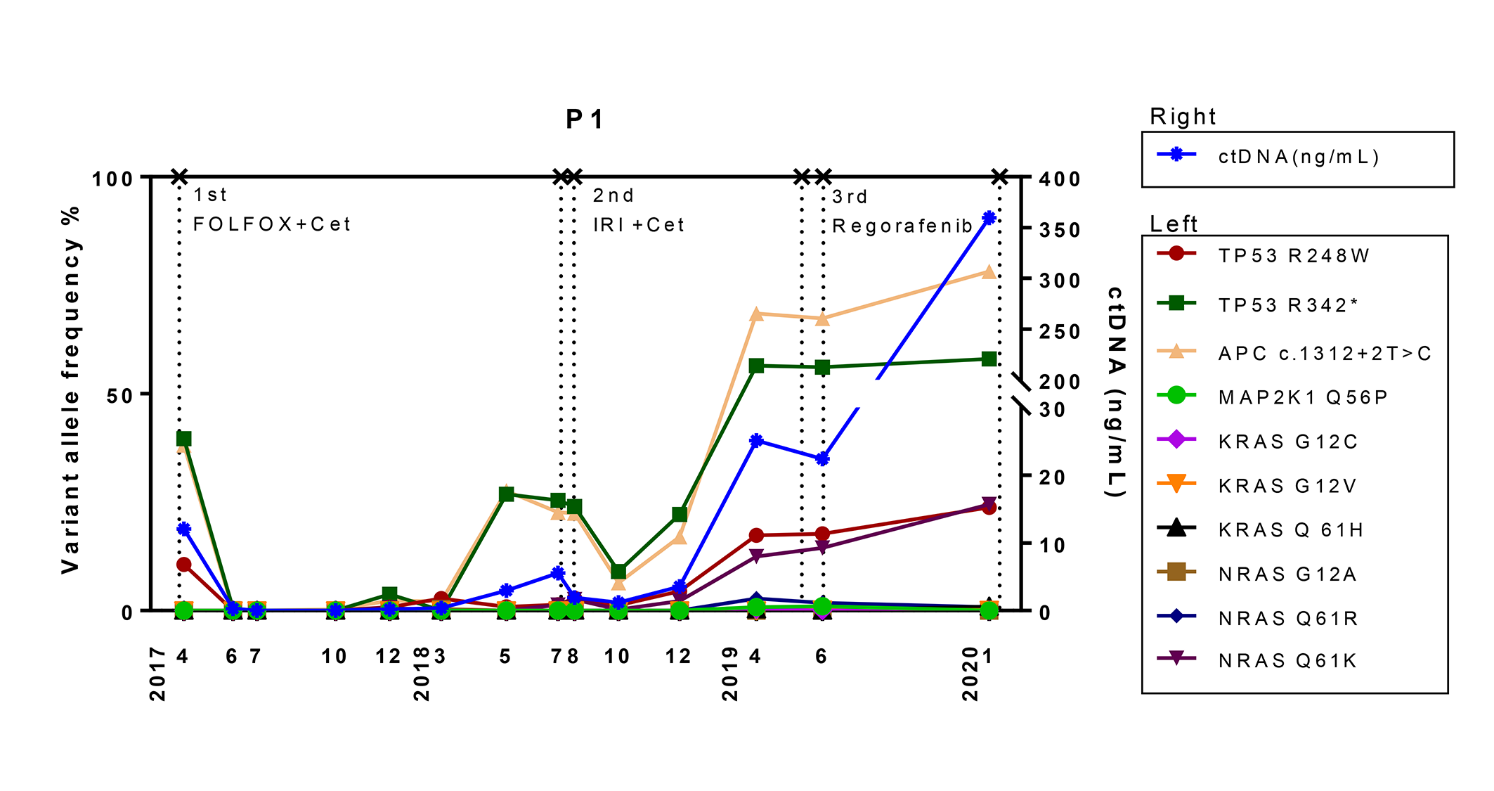
 **Supplementary Figure 2.** Clinical utility of longitudinal ctDNA testing in patient 1 (P1). ctDNA (ng/mL)=VAFmax*m(cfDNA)/Volume

## Supplementary Tables

**Table S1**. The list of 61 cancer-related genes.

| AKT1 | ALK | AR | ARAF | BCL2L11 | BRAF | BRCA1 | BRCA2 | CDK4 | CDKN2A |
| --- | --- | --- | --- | --- | --- | --- | --- | --- | --- |
| CYP2C19 | CYP2D6 | DPYD | EGFR | ERBB2 | ERBB3 | ESR1 | FGFR1 | FGFR2 | FGFR3 |
| GNA11 | GNAQ | GNAS | HRAS | IDH1 | IDH2 | JAK2 | JAK3 | KIT | KRAS |
| MAP2K1 | MAP2K2 | MDM2 | MET | MLH1 | MSH2 | MSH6 | MTOR | NRAS | NRG1 |
| NTRK1 | NTRK2 | NTRK3 | PALB2 | PDGFRA | PDGFRB | PIK3CA | PMS2 | POLD1 | POLE |
| PTEN | RAF1 | RB1 | RET | ROS1 | SMO | STK11 | TERT | TP53 | TPMT |
| UGT1A1 |  |  |  |  |  |  |  |  |  |

**Table S2.** The list of 65 cancer-related genes.

| AKT1 | ALK | APC | ARAF | ARID1A | ATM | BCL2L11 | BRAF | BRCA1 | BRCA2 |
| --- | --- | --- | --- | --- | --- | --- | --- | --- | --- |
| CCND1 | CD274 | CDK4 | CDK6 | CDKN2A | CTNNB1 | CYP2C19 | DDR2 | DPYD | EGFR |
| ERBB2 | ERBB3 | FBXW7 | FGF19 | FGFR1 | FGFR2 | FGFR3 | FLT3 | HRAS | JAK2 |
| KDR | KIT | KRAS | LRP1B | MAP2K1 | MAP2K2 | MET | MLH1 | MSH2 | MSH6 |
| MTOR | NF1 | NRAS | NTRK1 | NTRK2 | PDCD1LG2 | PDGFRA | PIK3CA | PMS2 | POLD1 |
| POLE | PTEN | RAF1 | RET | ROS1 | SMAD4 | SMO | SPTA1 | STK11 | TP53 |
| TPMT | TSC1 | TSC2 | UGT1A1 | VEGFA |  |  |  |  |  |
